# Mixed-effects models for GAW18 longitudinal blood pressure data

**DOI:** 10.1186/1753-6561-8-S1-S87

**Published:** 2014-06-17

**Authors:** Wonil Chung, Fei Zou

**Affiliations:** 1 Department of Biostatistics, University of North Carolina at Chapel Hill, 3101 McGavran-Greenberg Hall, Chapel Hill, NC 27599, USA

## Abstract

In this paper, we propose two mixed-effects models for Genetic Analysis Workshop 18 (GAW18) longitudinal blood pressure data. The first method extends EMMA, an efficient mixed-model association-mapping algorithm. EMMA corrects for population structure and genetic relatedness using a kinship similarity matrix. We replace the kinship similarity matrix in EMMA with an estimated correlation matrix for modeling the dependence structure of repeated measurements. Our second approach is a Bayesian multiple association-mapping algorithm based on a mixed-effects model with a built-in variable selection feature. It models multiple single-nucleotide polymorphisms (SNPs) simultaneously and allows for SNP-SNP interactions and SNP-environment interactions. We applied these two methods to the longitudinal systolic blood pressure (SBP) and diastolic blood pressure (DBP) data from GAW18. The extended EMMA method identified a single SNP on Chr5:75506197 (*p*-value = 4.67 × 10^−7^) for SBP and three SNPs on Chr3:23715851 (*p*-value = 9.00 × 10^−8^), Chr 17:54834217 (*p*-value = 1.98 × 10^−7^), and Chr21:18744081 (*p*-value = 4.95 × 10^−7^) for DBP. The Bayesian method identified several additional SNPs on Chr1:17876090 (Bayes factor [BF] = 102), Chr3:197469358 (BF = 69), Chr15:87675666 (BF = 43), and Chr19:41642807 (BF = 33) for SBP. Furthermore, for SBP, we found a single SNP on Chr3:197469358 (BF = 69) that has a strong interaction with age. We further evaluated the performances of the proposed methods by simulations.

## Background

Genome-wide association studies (GWAS) have been used for examining genetic variants associated with blood pressure and hypertension [1,2]. Because blood pressure changes over time, it is important to collect multiple blood pressure measurements to study time-dependent genetic effects. Genetic Analysis Workshop 18 (GAW18) data included systolic blood pressure (SBP) and diastolic blood pressure (DBP) measurements from a human whole genome sequencing (WGS) study [3]. The study was longitudinal, and the majority of participants had three measurements collected at approximately 5-year intervals. This paper proposes two mixed-effects models for GAW18 longitudinal SBP and DBP data. The first approach extends the EMMA method [4], an efficient mixed-model association-mapping algorithm. EMMA corrects for population structure and genetic relatedness using a kinship similarity matrix. We replace the kinship similarity matrix in EMMA with an estimated correlation matrix for the dependence structure of the multiple measurements from each individual. With this extended approach, hundreds of thousands or even millions of association tests can be performed efficiently. However, this approach tests only one single-nucleotide polymorphism (SNP) at a time and may have low power to map SNPs that interact with each other. Furthermore, it is not straightforward to tweak EMMA software for testing SNP by time interaction, an important question that can be addressed through longitudinal data. To address these concerns, we developed a Bayesian method based on the composite model space framework of Yi et al [5-7]. The proposed method fits multiple SNPs simultaneously. In addition, it allows for SNP-SNP interactions and SNP-time interactions.

## Methods

### Extended EMMA

For testing association between a given SNP and the longitudinal phenotype, we fit the mixed-effects model

(1) $$y_{i}=\mu_{i}+x_{i}^{e}\beta^{e}+x_{i}^{g}\beta^{g}+u_{i}+e_{i}\left(i=1,...,n\right)$$

where $y_{i}={\left(y_{i1},...,y_{in_{i}}\right)}^{T}$ is the $n_{i}\times 1$ phenotype vector of individual *i*; $\mu_{i}=\mu 1_{n_{i}}$ with *μ *being the grand mean and $1_{n_{i}}$ being the $n_{i}\times 1$ vector whose elements are all equal to 1; $x_{i}^{\text{e}}$ is the design matrix corresponding to nongenetic covariates (e.g., time), and $\beta^{\text{e}}$ is the associated nongenetic effects; $x_{i}^{g}$ is the numerically coded genotype of individual *i *and $\beta^{g}$ is the corresponding SNP effect. In the model, we assume random effect $u_{i}\sim N\left(\text{0},\sigma_{g}^{2}K_{i}\right)$ where $K_{i}$ is an $n_{i}\times n_{i}$ matrix, and random error $e_{i}\sim N\left(\text{0},\sigma_{e}^{2}I_{n_{i}}\right)$. The SNP effect can be tested as $H_{0}:\beta^{g}=0$ versus $H_{1}:\beta^{g}\neq 0$ via the likelihood ratio test. For GWAS or WGS data, this test needs to be performed with a large number of SNPs, which can be computationally intensive if we treat $K_{i}$s as the unknowns and estimate them jointly with the fixed effects. EMMA [4] is an efficient algorithm originally developed for GWAS data in which samples are potentially structured. EMMA models the structure effect via a similarity matrix. An R package that implements EMMA can either estimate the similarity matrix using genotype data or take any similarity matrix provided by users. We tweak EMMA for our purpose. We provide EMMA with the following similarity matrix $K=diag\left({\hat{K}}_{1},{\hat{K}}_{2},...,{\hat{K}}_{n}\right)$ where ${\hat{K}}_{i}$s are the estimated correlation matrices from model (1) in which $\beta^{g}$ is set to 0. The idea of estimating $K_{i}$s this way is not new and has been used in EMMAX [8], a fast version of EMMA. These estimates should be reasonable unless some SNPs have large effects, which is rare for most complex traits.

### Bayesian multiple QTL mapping

To further identify SNPs interacting with each other and with other nongenetic factors, such as time, we consider the following mixed-effects model

(2) $$\begin{array}{c} y_{i}=\mu_{i}+x_{i}^{\text{e}}\beta^{\text{e}}+x_{i}^{\text{g}}\beta^{\text{g}}+x_{i}^{\text{gg}}\beta^{\text{gg}}+x_{i}^{\text{ge}}\beta^{\text{ge}}+u_{i}+e_{i}\\ \;=\mu_{i}+x_{i}\beta +u_{i}+e_{i}\left(i=1,...,n\right) \end{array}$$

where $x_{i}\left[=\left(x_{i}^{\text{e}},x_{i}^{\text{g}},x_{i}^{\text{gg}},x_{i}^{\text{ge}}\right)\right]$ is the design matrix corresponding to nongenetic factors, *p *putative SNPs, two-way interactions between *p *SNPs (resulting in total of *p*(*p*−1)/2 terms) and other selected SNP-environment interactions (for GAW18 data, we consider *p *SNP-age interactions); $\beta \left[={\left(\beta^{\text{e}T},\beta^{\text{g}T},\beta^{\text{gg}T},\beta^{\text{ge}T}\right)}^{T}\right]$ is the vector of all fixed effects. We define $\mu_{i}$ the same way as in model (1). The random effects ***u**_i _*and ***e**_i _*are also assumed to follow the same distributions as described in model (1). Model (2) includes the effects of all putative SNPs; thus, the number of such effects can be large. To identify SNPs associated with the trait of interest, we use a Bayesian variable selection procedure in which we use a set of latent binary variables $\gamma_{k}$(k=1,...,q) to indicate which of the *q *genetic effects (be they main genetic effects, epistasis effects and/or SNP by environment interactions) are associated $\left(\gamma_{k}=1\right)$ or not associated $\left(\gamma_{k}=0\right)$ with the trait.

As in model (1), we assume matrix $K_{i}$ is known. We apply the Cholesky decomposition to $K_{i}$ such that $K_{i}=M_{i}M_{i}^{T}$ where $M_{i}$ is the $n_{i}\times n_{i}$ lower triangular Cholesky decomposition matrix of $K_{i}$. Then model (2) can be reparameterized as $y_{i}=\mu_{i}+x_{i}\beta +\sigma_{g}M_{i}b_{i}+e_{i}$ where $b_{i}={\left(b_{i1},...,b_{in_{i}}\right)}^{T}\sim N\left(\text{0},I_{n_{i}}\right)$. We use the same prior distributions for *μ*, *β*, $\gamma ={\left(\gamma_{1},...,\gamma_{q}\right)}^{T}$, and $\sigma_{e}^{2}$ in Yi et al [7]. We set the prior of $\sigma_{g}$ to $N^{+}\left(m_{g0},s_{g0}^{2}\right)$, where $N^{+}\left(\mu_{0},\sigma_{0}^{2}\right)$ is the positive truncated normal density with mean $\mu_{0}$ and variance $\sigma_{0}^{2}$, and both $m_{g0}$ and $s_{g0}^{2}$ are prespecified hyperparameters. The proposed method has been implemented upon the widely used R package, R/qtlbim [9] for these GAW18 longitudinal data.

## Results and discussion

### GAW18 data

The GAW18 data included 849 individuals with both phenotype and imputed genotype data from 20 extended pedigrees. Each sample was measured multiple times on their blood pressures over approximately 5-year intervals. Among these 849 individuals, 139 were genetically unrelated and were measured for age, sex, medication use, smoking status, and blood pressure. Our analysis was restricted to the 139 unrelated individuals. The number of SBP and DBP ranged from one to four for each sample. WGS data provided by the GAW18 data had 8,348,674 SNPs from odd numbered autosomes. All SNPs provided passed the initial quality control checking, but among 2,796,608 SNPs with minor allele frequency (MAF) greater than 0.05, 17,463 of them failed Hardy-Weinberg equilibrium (HWE) test (with *p*-value<0.05/2,796,608, a Bonferroni corrected genome-wide threshold). We removed all SNPs with MAFs less than 0.05 plus those not passing the HWE test, resulting in 2,779,145 SNPs for the subsequent analyses.

To check population outliers and potential population substructure, we generated a subset of SNPs that are not in high linkage disequilibrium (LD) with each other (i.e., $r^{2}<0.5$) and performed the multidimensional scaling (MDS) analysis in PLINK [10]. Pairwise scatter plots of the top four MDS scores showed that the 139 individuals are homogeneous in terms of their ethnicities. However, two samples, T2DG0400207 and T2DG0400247, have an estimated IBD value of 0.3 between them, indicating that they are likely related. In our analysis, we retained all 139 samples because the number of putatively related samples is small and their inclusion should have a negligible effect on our analysis results.

**Figure 1:**
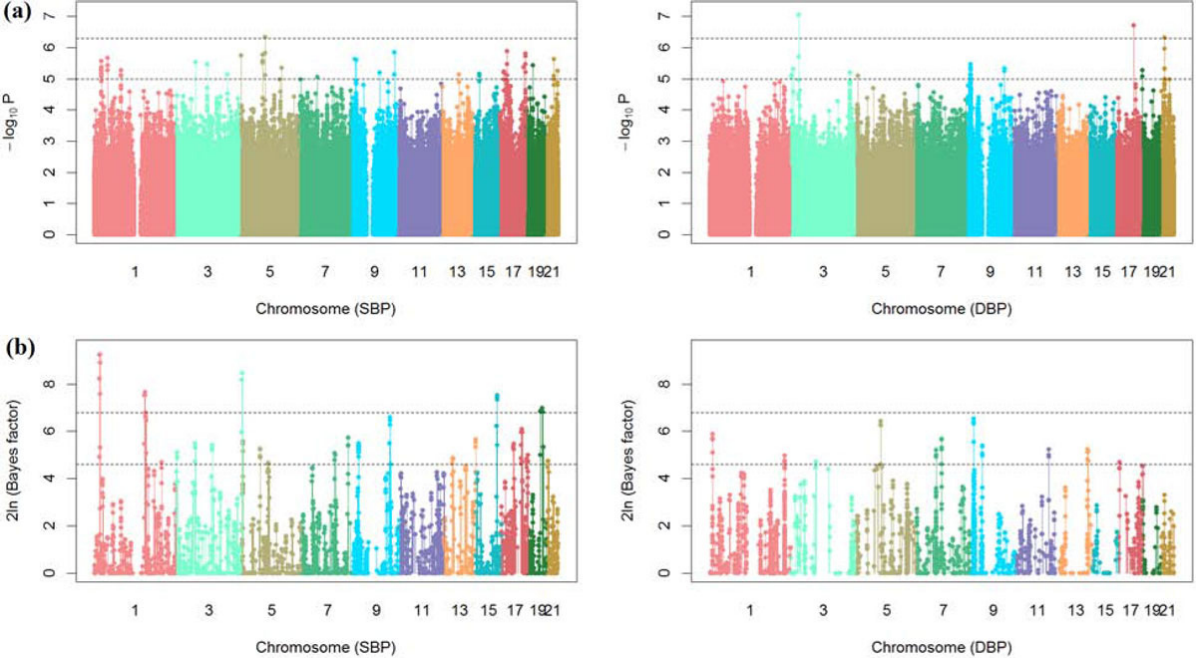
**Manhattan plots on Genetic Analysis Workshop 18 (GAW18) longitudinal data**. (a) Manhattan plots of -log_10 _(*p*-value) for systolic blood pressure (SBP) and diastolic blood pressure (DBP) from the extended EMMA. The two dashed horizontal lines represent the genome-wide thresholds for suggestive (*p*-value = 10^−5 ^and significant (*p*-value = 5 × 10^−7^) associations. (b) Manhattan plots of 2 in (BF) for the proposed Bayesian method. Two dashed horizontal lines represent the genome-wide thresholds for moderate (BF = 10) and strong (BF = 30) associations.

We applied the two proposed procedures to these filtered GAW18 data on the two log-transformed phenotypes, log(SBP) and log(DBP). Five covariates (age, age^2^, sex, medication use, and smoking status) were included for analyses. We fitted these data with different covariance matrices in SAS 9.2 and selected the spatial power covariance structure for the downstream analysis based on the AIC criteria. Specifically, we let $cov\left(y_{ij},y_{ij^{\prime }}\right)=\sigma^{2}\rho^{d_{i,jj^{\prime }}}$, where $d_{i,jj^{\prime }}$ is the time distance between the *j*th and $j^{\prime }$th examinations for individual *i*. After obtaining the parameter estimate of *ρ*, $\hat{\rho }$ from model (1) with $\beta^{g}=0$, we substituted the kinship matrix *K *in EMMA by $K=diag\left({\hat{K}}_{1},{\hat{K}}_{2},...,{\hat{K}}_{n}\right)$ where ${\hat{K}}_{i}=\left\{{\hat{\rho }}^{d_{i,jj^{\prime }}}\right\}$. Figure 1(a) displays the Manhattan plots of the two phenotypes from the extended EMMA model. For SBP, one SNP on Chr5:75506197 ($P=4.67\times 10^{-7}$) reached the genome-wide significance ($p-\text{value}<5\times 10^{-7}$, a cutoff suggested by Burton et al [11]). For DBP, three SNPs on Chr3:23715851 ($p-\text{value}=9.00\times 10^{-8}$), Chr17:54834217 ($p-\text{value}=1.98\times 10^{-7}$) and Chr21:18744081 ($p-\text{value}=4.95\times 10^{-7}$) exceeded the genome-wide significance.

Because of the limited sample size, it is not feasible to include all available SNPs in our Bayesian analysis. For each phenotype, we selected a list of 3000 top-ranked SNPs that are not highly correlated with each other (with correlation < 0.95 to avoid multicollinearity) from the extended EMMA for the Bayesian analysis. We applied this Bayesian method with the same covariates used in the extended EMMA method. For all analyses, the MCMC algorithm ran for $4\times 10^{5}$ iterations after the first 1000 burn-in iterations were discarded. The chain was then thinned for every 40 iterations, yielding $10^{4}$ MCMC samples for the posterior analysis. Based on the posterior inclusion probability of each SNP, the Bayes factor (BF) (see [6,7] for details) was estimated and used to judge the importance of each SNP. Figure 1(b) shows the Manhattan plots of 2ln(BF) for the combined genetic effects of each SNP, which include the main effects, epistasis effects, and SNP-age interactions. We found several additional SNPs with strong signals (BF >30 as suggested by Yandell et al [12]) on Chr1:17876090 (BF = 102), Chr3:197469358 (BF = 69), Chr15:87675666 (BF = 43), and Chr19:41642807 (BF = 33) for SBP. No new SNPs were found for DBP. For SBP, we found one SNP located on Chr3:197469358 (BF = 69) has a strong interaction with age.

### Simulations

**Figure 2:**
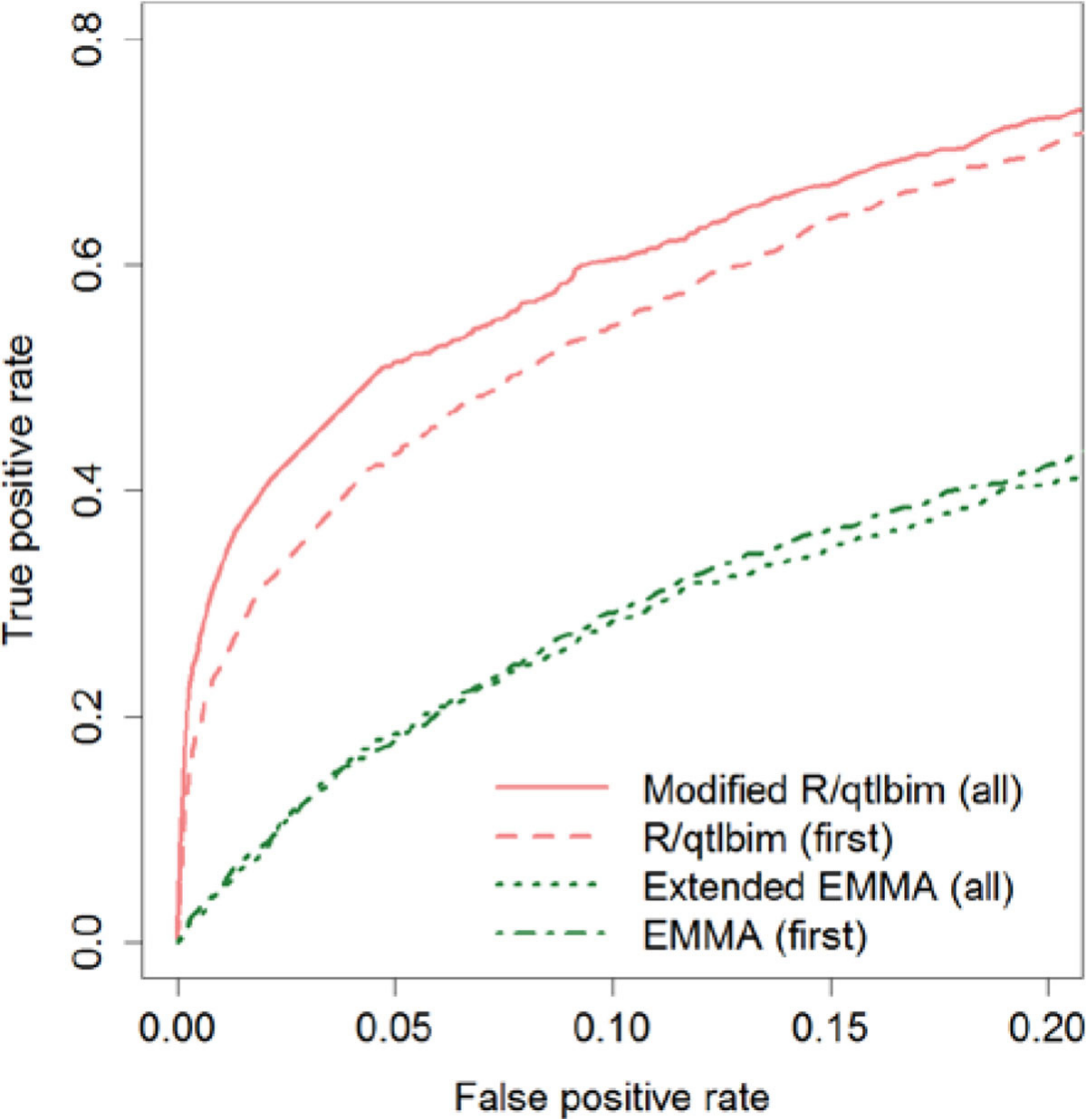
**Receiver operating characteristic curves on simulated data**. Solid line represents proposed Bayesian method (i.e., modified R/qtlbim) on all data; dashed line, R/qtlbim on first time point data; dotted line, extended EMMA on all data; and dot-dashed line, EMMA with first time point data.

To evaluate the performances of the proposed methods, we conducted the following simulations. From the 3000 top-ranked SBP SNPs previously selected, we randomly picked up 10 of them that are at least 10 Mb apart as causal SNPs and called them $SNP_{1}$,...,$SNP_{10}$. Among the 10 causal SNPs, we let 7 of them have only main effects, 2 have an epistasis effect, and 1 have an SNP-age interaction. The estimated correlation matrix $diag\left({\hat{K}}_{1},{\hat{K}}_{2},...,{\hat{K}}_{n}\right)$ along with $\sigma_{g}^{2}=0.8$ was used to simulate the random effects $u_{i}$s. We set $\sigma_{e}^{2}$ to 1. Specifically, we simulated data according to the following model: $y_{i}=\left(SNP_{1i}+...+SNP_{7i}+SNP_{8i}\cdot SNP_{9i}\right)1_{n_{i}}+SNP_{10i}\cdot age_{i}+u_{i}+e_{i}$ where $u_{i}\sim N\left(\text{0},\sigma_{g}^{2}K_{i}\right)$ and $e_{i}\sim N\left(\text{0},\sigma_{e}^{2}I_{n_{i}}\right)$. A total of 100 simulations were performed. We compared the two proposed methods with each other and with two other existing methods, the original EMMA and R/qtlbim methods. The last two methods only work for univariate data, so we applied them to the simulated data with only first-time measurements used. To make the methods comparable, we generated the receiver operating characteristic (ROC) curve for each method as described later. For a given cutoff of *p*-value or BF, we calculated the true and false positive findings as follows: a significant finding is claimed to be a true positive finding if it is located less than 1 Mb from any one of the simulated causal SNPs; otherwise the finding is false. The ROC curves with the false-positive rate less than 0.2 are presented in Figure 2. Intuitively, our two methods that used all available data are more powerful than their corresponding univariate analysis methods that only used the first-time data. Furthermore, the Bayesian method is more powerful than the extended EMMA as expected because (a) the Bayesian model allows for SNP-SNP and SNP-age interactions, which are totally ignored by the extended EMMA, and (b) the Bayesian model jointly model multiple SNPs, but the extended EMMA only tests one SNP at a time.

## Conclusions

In this paper, we developed two mixed-effects models for the GAW18 longitudinal blood pressure data. The first approach extends the EMMA method. We replace the kinship similarity matrix in EMMA with an estimated correlation matrix for dealing with the dependent structure of the repeated measurements. The second approach is a Bayesian method that models multiple SNPs simultaneously and allows for SNP-SNP interactions and SNP-time interactions. The advantages of the Bayesian method have been clearly demonstrated by our simulations. Both methods are currently developed for unrelated samples. The GAW18 data contained extended pedigrees. Ideally, we should use all available data in our analysis. What complicates the analysis on longitudinal pedigree data is that both the correlation structure of the repeated measurements and the familial correlation structure of related individuals should be considered. We are currently extending the two proposed methods for the GAW18 pedigree data. Furthermore, for both our analyses, we assume that the covariance matrix is known up to a constant. For the Bayesian model, this assumption can be relaxed and we are developing a semiparametric approach where the covariance matrix is assumed unknown. We estimate the unknown covariance matrix with a modified Cholesky decomposition [13]. Last, our Bayesian model for GWAS data relies on a set of preselected putative SNPs. How to select a good set of putative SNPs, especially those with low marginal effects but high interactions with other SNPs or environmental factors is challenging and deserves further investigations.

## Competing interests

The authors declare that they have no competing interests.

## Authors' contributions

WC developed, implemented methods, performed statistical analysis, and drafted the manuscript. FZ designed the study, directed the research, revised the manuscript critically, and gave final approval for publication. All authors read and approved the final manuscript.
